# Autochthonous Leishmaniosis in Dogs, Cats, Horses, and Wildlife in the United States of America

**DOI:** 10.3390/microorganisms14040930

**Published:** 2026-04-20

**Authors:** Chaoqun Yao, Yi Yang, Aifang Du

**Affiliations:** 1 Department of Biomedical Sciences, One Health Center for Zoonoses and Tropical Infectious Diseases, Ross University School of Veterinary Medicine, Basseterre P.O. Box 334, Saint Kitts and Nevis; 2 Institute of Preventive Veterinary Medicine, College of Animal Sciences, Zhejiang University, Hangzhou 310058, China; yangyi0607@zju.edu.cn (Y.Y.); afdu@zju.edu.kn (A.D.)

**Keywords:** autochthonous leishmaniosis, *Leishmania*, USA, transmission, dog, cat, horse, wildlife

## Abstract

Leishmaniasis is endemic in 99 countries worldwide, including the United States of America (USA). Its causative pathogens, *Leishmania* spp. have been detected in both humans and animals within the USA. Lately, we have comprehensively reviewed autochthonous leishmaniasis in humans in this region. Animals play a pivotal role in maintaining its endemicity in some endemic areas and regions, for example, dogs in Brazil and the Mediterranean. In this review, we thoroughly examine autochthonous leishmaniosis in dogs, cats, horses, and wildlife in the USA, and we synthesize integration across species, transmission pathways, the crucial roles the animals play, and the potential risk they pose to humans. The information is essential for public health and for the effective control and management of leishmaniasis through expanding reservoir and vector surveillance using One-Health approaches in the USA.

## 1. Background

The United States of America (USA) is designated as an endemic country with leishmaniasis by the World Health Organization (WHO, https://apps.who.int/neglected_diseases/ntddata/leishmaniasis/leishmaniasis.html) (accessed on 11 April 2026). The disease manifests in humans in three major forms: cutaneous leishmaniasis (CL), visceral leishmaniasis (VL) and mucocutaneous leishmaniasis [1,2]. There are 0.6–1.0 million new CL cases and 50–90 thousand VL cases worldwide annually (WHO). Specifically, all 89 cases of autochthonous leishmaniasis in humans in the USA are CL, reported in Texas, Oklahoma, Arizona and North Dakota [3]. In addition to humans, autochthonous leishmanioses have been reported in the USA in dogs, cats, horses and even wildlife. The latter includes Southern Plains woodrats (*Neotoma micropus*), Eastern woodrat (*N. floridana*), White-throated woodrat (*N. albigula*) and Texas deermice (*Peromyscus attwateri*) [4,5]. Hence, this review is on autochthonous leishmaniosis in dogs, cats, horses, and wildlife, which complements a late publication on human leishmaniases originating in the USA [3].

## 2. Case Identification

### 2.1. Criteria for Autochthonous Leishmaniosis

A confirmed case of autochthonous leishmaniosis in dogs, cats and horses must meet the same criteria as those outlined for human cases as previously described [3]. These are: (1) The animals must have no travel history outside of the USA in their lifetime; (2) The animals should not have traveled abroad to an endemic area(s) within five years preceding disease onset. The time window eliminates the majority of, if not all possible, imported cases since the incubation period for *L. infantum* VL in dogs is seldom longer than five years.

### 2.2. Identification of Leishmania spp.

For *Leishmania* spp. Identification, molecular techniques are often required since morphology itself cannot discriminate between species even by electron microscopy (EM). These include isoenzyme profile, PCR, DNA sequencing, and metagenomic next-generation sequencing [3].

## 3. Autochthonous Leishmaniosis

### 3.1. Autochthonous Cases in Dogs

During the necropsy of a seven-year-old female Foxhound with a history of alopecia and anemia, *Leishmania* amastigotes were found in macrophages in the bone marrow smears and impressions of the prescapular lymph node. Further, high titers of antibodies to promastigotes of *L*. *donovani* and *L*. *tropica* were detected, although negative for antibodies to *L*. *braziliensis* promastigotes and *T*. *cruzi* epimastigotes. The dog had never traveled beyond the 150-mile radius of Oklahoma City, Oklahoma, its entire life, confirming an autochthonous leishmaniosis case in Oklahoma State and the USA [6]. The parasite species was later determined to be *L*. *infantum* by isoenzyme profile [7]. Since then, 42 autochthonous canine cases have been reported in the USA (Table 1). Importantly, the *Leishmania* sp. identified in all 11 reported cases is *L*. *infantum*. Among 16 cases with known breeds, the breeds included: Basenji—1, Beagle—2, Boxer—1, Doberman Pinscher—1, Foxhound—7, Foxhound-Treeing Walker Coonhound cross—1, Golden Retriever—1, Newfoundland—1 and Toy Poodle—1. They were five females and six males reported with five of unknown sex. The recorded ages of nine dogs range from 1 year to 11 years (Table 1).

Cross-sectional surveys have also been carried out in the USA. Among 112 Foxhounds in the index Foxhound kennel in Dutchess County, New York, 33 (29.5%) tested positive for antibodies to *Leishmania* spp. by Indirect Immunofluorescence Assay (IFA). Further, 26 (23.2%) had confirmed infections by the microscopic identification of *Leishmania* amastigotes and/or PCR amplification of the *Leishmania* sp. DNA [8]. In a cross-sectional serological survey from April 2000 to December 2003, more than 12,000 canine sera were tested for antibodies to *Leishmania* spp. by IFA. These were mainly from Foxhounds owned by members of The Masters of Foxhounds Association of America across 35 U.S. states. The serum-positive dogs were followed-up by a parasitological confirmation of the parasite in tissues, such as bone marrow, and parasite culture. Cultured parasites were then identified to species using isoenzyme profile performed by the Reference Center in Rome, Italy. Foxhounds in 18 states were found infected with *L. infantum*. The states with infected Foxhounds included Alabama, Connecticut, Georgia, Iowa, Illinois, Indiana, Kentucky, Maryland, Michigan, Missouri, North Carolina, New Jersey, New York, Ohio, Pennsylvania, South Carolina, Tennessee, and Virginia [9]. Interestingly, no infections were found in other canine breeds nor were humans who worked closely with positive Foxhounds found to be infected. Possible transmission routes among Foxhounds were proposed as direct dog-to-dog transmission, which may occur through biting, blood transfusions, and breeding [9]; this was later confirmed and will be illustrated in a section entitled “transmission routes”. In another cross-sectional serological survey of dogs across the USA, 957 sera collected between January 2000 and August 2001 were tested by IFA for antibodies to *L. donovani* promastigotes. All the dogs sampled happened to be non-Foxhounds, and all tested negative except two. One was a 2-year-old male Bullmastiff from Texas; the other was a 2.5-year-old neutered male Greyhound from New York. Both dogs tested negative by PCR for *Leishmania* spp. DNA. They also tested positive for antibodies to *Trypanosoma cruzi* by both IFA and radioimmunoprecipitation assay (RIPA) [10]. It is very likely that both dogs were false positive due to *T. cruzi* infections since antibodies to *Leishmania* spp. do not yield positive results in *T. cruzi*-RIPA [9,11]. Therefore, all 957 non-Foxhounds surveyed in the study had not previously been exposed to *Leishmania* spp.

A retrospective study was carried out among dogs in the USA and Canada between January 2006 and May 2019. They were tested for leishmanial infections by IFA using *L. infantum* as antigens, PCR or both. Among 1961 dogs included in the study, 125 were found positive, with only eight from Canada. Travel history of 69 dogs was provided by primary veterinarians of the cases. Only nine cases were determined to be autochthonous, including four Foxhounds and five non-Foxhounds. All non-Foxhounds were from the USA (AZ—3, CA—1 and WA—1); only two of four Foxhounds were confirmed to originate from the West South-Central region of the USA (AR, LA, OK, TX) (Table 1). The remaining sixty dogs had traveled to endemic countries including Spain, Italy and Greece in the last five years [12].

Further, surveillance, both active and passive, was carried out among U.S. hunting hounds for vertical transmission of canine leishmaniosis over a nine-year span between 2007 and 2015 using qPCR optimized to detect the kinetoplast DNA of *L. infantum*. The numbers of each cohort were 446 and 560 dogs, respectively. No statistical differences in prevalence and incidence were found between active and passive cohorts. The prevalence and incidence ranged from 0% to 68% with the average percentage of positive tests per year being 26.7% and 20.2% in the active and passive cohorts, respectively [13].

**Figure 1:**
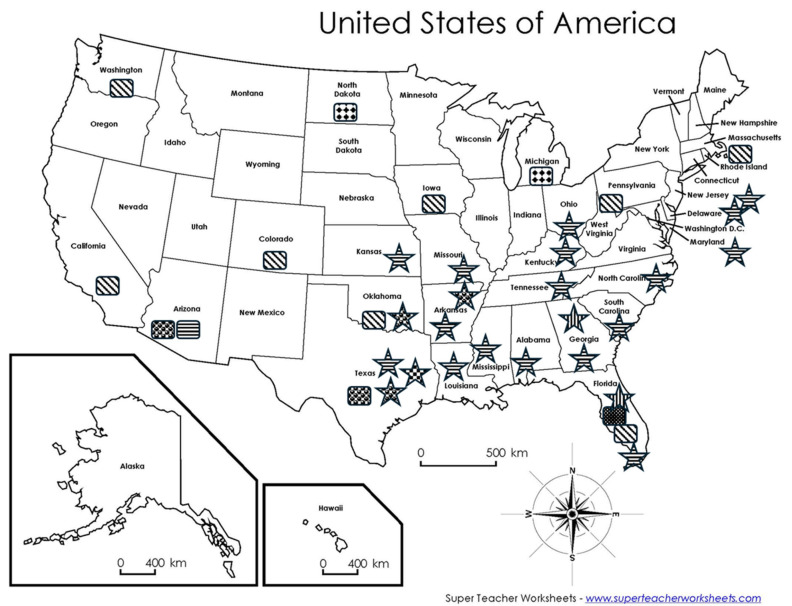
The distribution of *Leishmania* spp. parasites and capable sand fly vectors indicates risk levels of individual states for leishmaniasis endemicity in the USA. Reproduced from [3]. *Leishmania* spp: *L. mexicana*: 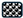; *L. infantum*: 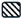; *L. donovani*: 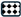; *L. ellisi*: 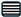; *L. martiniquensis*: 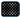; sand fly, *Lutzomyia* species: *Lu. anthophora*: 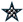; *Lu. cruciate*: 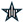; *Lu. diabolica*: 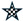; *Lu. shannoni*: 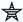.

Collectively, canine autochthonous infections by *Leishmania* spp. have occurred in several U.S. states, and several canine breeds have been affected, especially Foxhounds (Figure 1 and Table 1). Interestingly, the parasite species in dogs is exclusively *L. infantum*, a species causing VL. This is very different from the autochthonous cases in humans [3] and in cats as described later, which is *L. mexicana* causing CL. In the presence of capable sand fly vectors, *L. infantum* can be transmitted between humans and dogs [14]. The interesting canine case in Maryland might showcase this transmission although it is currently under debate in the USA. An 11-year-old male cryptorchid Toy Poodle of no travel history outside of the state of Maryland or the Washington, DC, area was diagnosed with leishmaniosis [15]. The transmission must have occurred in Maryland, which has the capable sand fly vector, *Lutzomyia* (*Lu.*) *shannoni*. Further, the owner of this diseased dog had traveled to Greece, an *L. infantum* endemic country, every two years during ownership of the dog. Unfortunately, the owner’s status of *Leishmania* sp. infection was not determined [15]. Hence, those *L. infantum*-positive canine hosts pose risks to humans in addition to dogs themselves. In order to reduce sources of leishmanial infections, dogs should be properly treated once diagnosed with VL. An alternative, yet remote option is euthanasia when poor prognosis is anticipated, which might be one of the reasons that a high proportion of diseased dogs listed in Table 1 underwent euthanasia.

**Table 1:** Autochthonous canine cases of leishmaniosis in the USA (open cells indicate no data available).

Year	State	Age	Sex	Breed	Clinical Signs	Diagnostic Methods *	*Leishmania* sp. ^‡^	Treatment (/F/H/LTF) ^¥^	References
1980	OK	7	F	Foxhound	Alopecia, muscle atrophy, anemia	M; S; IP	*L. infantum*	None, euthanasia	[6,7]
1978–1983	^a^ OK & KS			Foxhound		M: S; C			[7]
1988	OH	6	M	Foxhound	Anorexia, weight loss, pyrexia, panuveitis, splenomegaly	M		Death	[16]
1989	^b^ MI			Foxhound	anorexia, listlessness, vomiting	M; C; S; IP			[7]
1991	TX	<1	F	Basenji	Fever, diarrhea, anemia,	M; S		Diminazene, ketoconazole, death	[17]
2000	MD	11	M	Toy Poodle	Depression, lethargy, weight loss, hepatosplenomegaly	M; S		Sodium stibogluconate, euthanasia	[15]
2000	PA	5	M	Newfoundland		M; S; IP	*L. infantum*		[7]
2001	FL FL		F	Beagle	Alopecia; lameness	M; S; PCR M; S; PCR	*L. infantum* *L. infantum*		[7]
2001	MA	3	F	Doberman Pinscher		M; C	*L. infantum*		[7]
2007	IA	1 1	F M	Foxhound Foxhound	Seropositive to *Leishmania* Seropositive to *Leishmania*	M; S; I M; S; C; PCR	*L. infantum* *L. infantum*	Euthanasia Euthanasia	[18]
2008	CO	1	M	Foxhound-Treeing Walker Coonhound cross	Diarrhea, weight loss, dermatologic lesions, mild hepatosplenomegaly	M, S, PCR	*L. infantum*	Euthanasia	[19]
2017	CA	1	M	Boxer	granulomatous cutaneous lesions, enlarged prescapular lymph node and anemia, cutaneous lesions	M, S, PCR, D	*L. infantum*	Marbofloxacin and allopurinol (F), euthanasia	[20]
2006–2019	^c^ AZ WA ^d^ N/A			Beagle Golden retriever Foxhound	Kidney failure and weight loss Anemia, weight loss and anorexia	S M, S; PCR S; PCR	*L. infantum* *L. infantum*		[12]

*: C: culture; D: DNA sequencing; I: immunohistochemistry; IP: isoenzyme profile; M: microscopy of amastigotes; S: serology; PCR: polymerase chain reaction. ^‡^: All are undetermined *Leishmania* sp. unless specifically indicated. ^¥^: F: failure; H: healed; LTF: lost to follow. ^a^: Two cases from OK, six from KS, an additional 5 likely from OK; ^b^: seven cases; ^c^: three cases; ^d^: four cases were reported without clear identification of states (N/A: not available); two were from the West South-Central region (AR, LA, OK, TX).

### 3.2. Autochthonous Cases in Cats

The first reported case of autochthonous leishmaniosis in cats in the USA is quite unique. The patient had been followed-up for more than seven years since the initial onset of clinical signs of CL. The patient was a male domestic, six-year-old (four in the original report) long-haired cat in 1984 living in Uvalde, Texas. The cat presented to a veterinarian due to several skin lesions on the left pinna, four of which were large with a diameter of 5–7 mm. The patient was diagnosed with CL by microscopy including EM, and the parasite was determined to be *L. mexicana* by isoenzyme profile. A radical pinnectomy was performed to minimize the risk of the ear serving as a reservoir [21]. In the following seven years, the same cat repeatedly had a few more episodes of skin lesions. First, in 1987, three years after the original diagnosis of leishmaniosis and pinnectomy, a skin lesion appeared on the pinnectomized ear. The following year a cutaneous lesion of 1.5 cm in diameter was found on the cat’s muzzle followed by a few smaller lesions. The cat was then treated topically with paromomycin solution without resolution of the lesions. In November 1990, an additional pink lesion was found on the right mucosa of the nasal septum. Further, the cat had never tested serologically positive for feline immunodeficiency virus or feline leukemia virus. Amastigotes had been repeatedly detected during those years of treatment in the biopsied lesions on the head but not among other tissues. The cat was euthanized at 13 years old due to lymphosarcoma [22]. Necropsy demonstrated the absence of leishmanial parasites in visceral organs. This chronologically well-documented case of CL followed the progressive prognosis of diffuse cutaneous leishmaniosis in a feline host in the USA.

Ten autochthonous cases of feline leishmanioses have been reported to date in the USA. They include seven males and three females. The available ages of nine cats range from one to 11 years old with a median of 4.5 years (Table 2). Further, sand flies were trapped on the residential property of the owner of an *L. mexicana*-infected cat in Bryan, Texas. Three female sand flies were caught and identified by PCR and DNA sequencing of *cox1* and internal transcribed spacer (ITS)-2 as *Lu. shannoni* (two) and *Lu. anthophora* (one) [23].

### 3.3. Autochthonous Cases in Horses

In addition to pet dogs and cats, *Leishmania* spp. infections have also been reported in horses. There are two reports on horses infected by *Leishmania* spp.; both are from Florida. The first one was a 10-year-old Morgan mare with ulcerated lesions on the ear pinna. A 6 × 3 cm lesion was present on the inside pinna, and three additional nodules of approximately 1 cm in diameter were located on the outside of the left pinna. There were also multiple 1–3 cm nodules on neck, shoulder and withers. The animal had never traveled outside of the eastern USA. A confirmed diagnosis and species identification of *L. siamensis* were made by microscopy revealing amastigotes in biopsied tissue, PCR and DNA sequencing of ITS1 [25]. *Leishmania siamensis* is now considered as a synonym of *L. martiniquensis* [26,27]. The second case was a 10-year-old neutered male Quarter horse. Ulcerated lesions were found on both pinnae. PCR and DNA sequencing targeting ITS1 determined the parasite species to be *L. martiniquensis* [28]. *Leishmania martiniquensis* is a new species that causes CL in humans on the island of Martinique, West Indies, in the Caribbean [29]. Interestingly, this parasite has also been found on the other side of the globe in Lamphun province, Thailand, where it was firmly determined to be the etiological pathogen of a human VL case [1,27]. Until now, no sand fly vectors have been incriminated as capable vectors of *L. martiniquensis* in Florida. Nevertheless, risk for human infection by this parasite exists in the state due to the following: (1) There have been two autochthonous cases in horses. (2) The parasite infects humans in other regions of the world leading to both CL and VL. (3) Capable sand fly vectors such as *Lu*. *shannoni* and *Lu*. *cruciate* for other *Leishmania* spp. are found in the state (Figure 1) although their roles in the transmission of *L. martiniquensis* remain uncertain.

### 3.4. Autochthonous Cases/Infections in Wildlife

Woodrats have been confirmed to be infected by and to serve as reservoirs for *L. mexicana* in the USA. The species implicated include both Southern Plains woodrats (*N. micorplus*) and Eastern woodrat (*N. floridiana*) in Texas, and White-throated woodrat (*N. albigula*) in Arizona [5,30]. Three female Southern Plains woodrats were collected in January 1990 in Zavala County, Texas. One was found infected with *L. mexicana* by cell culture followed by isoenzyme profiling [31,32]. One Eastern woodrat caught in January 2001 in Grimes County, Texas, had lesions on both ears and swollen feet. It was found infected with *L. mexicana* by PCR targeting species-specific kinetoplast DNA [33]. In a dynamic study of mark–release of 192 including 35 recaptures of Southern Plains woodrats in southern Texas from October 1989 to October 1992, fourteen were culture-positive, which presented an annual prevalence of 5.6–27% [34]. Twenty-eight White-throated woodrats (18 along the Arivaca Creek and 10 in the El Cadaza Refuge) in Pima County, Arizona were trapped and biopsied in each ear in September and October 1998. The biopsied tissues were subjected to cell cultures and PCR targeting *Leishmania* kinetoplast DNA minicircles. Two positive cultures were identified as *L. mexicana* by isoenzyme profiling [30].

Twenty sylvatic mammals were trapped in October 2011 from Mason County, Texas. These included six raccoons (*Procyon lotor*), one White-throated woodrat, three hispid cotton rats (*Sigmodon hispidus*), four white-ankled deermice (*Peromyscus pectoralis*), three white-footed deermice (*P. leucopus*), two Texas deermice (*P. attwateri*), and one Piñon deermouse (*P. truei*). They were all tested for *L. mexicana* by PCR and DNA sequencing. One adult male Texas deermouse was found to be positive [35]. Hence, Texas deermice likely serve as a natural reservoir for *L. mexicana* in addition to woodrats. Furthermore, it is plausible to test synanthropic rodents such as species of the genus *Rattus* and *Mus musculus* for *Leishmania* spp., which could represent a valuable surveillance strategy, particularly when using biological samples obtained from pest control programs.

A cross-sectional serological survey was performed for wild canids using a commercially available ICT kit for the domestic dog. The wild canine sera included in the study were derived from 11 foxes (*Vulpes vulpes*) and 240 coyotes (*Canis latrans*) collected in Philadelphia, Pennsylvania, and from 16 coyotes in Tennessee. Five samples from Philadelphia tested positive, including one fox and four coyotes. The prevalence was 9.1% (1/11) and 1.7% (4/240), respectively. All 16 samples from Tennessee tested negative [36]. This study raises concerns that wild canids such as foxes and coyotes are exposed to *Leishmania* spp. and may serve as natural reservoirs in the USA. Notably, these wild canids were not tested for *T. cruzi* using RIPA to rule out cross reactions. Twenty-seven sera collected from coyotes in central Georgia were tested for antibodies to *Leishmania* spp. None tested positive [37]. In a cross-sectional serological survey in North Carolina, 26 gray foxes (*Urocyon cinereoargenteus*) and two coyotes were found to be negative for antibodies to *L. infantum* [38]. Taken together, whether wild canids such as foxes and coyotes are infected with and likely serve as reservoirs for *Leishmania* spp. in the USA remains to be confirmed. Studies with larger sample sizes to detect *Leishmania* parasites from various individual states are warranted to get a better picture of wild animal reservoirs for *Leishmania* parasites. An additional source of wild animals are zoo animals. Monitoring autochthonous infections and transmission of *Leishmania* spp. through wildlife in zoos could be feasible, provided that care is taken to rule out imported cases.

## 4. *Leishmania* spp. in the USA

In all, five *Leishmania* species have been confirmed in the USA by molecular techniques including isoenzyme profile, PCR, and DNA sequencing [3]. Interestingly, parasites identified from autochthonous canine cases are exclusively *L. infantum* (Table 1); the parasites in feline cases are all *L. mexicana* (Table 2); and the parasites infecting horses are *L. martiniquensis* [25,28]. All human cases in the USA are caused by *L. mexicana* except two cases, one by *L. donovani* complex and the other by *L. ellisi* [3,39]. The geographical distribution of the five *Leishmania* spp. is presented in Figure 1. Among them, *L. mexicana* is the leading cause of CL in domestic cats as well as in wildlife—namely, the woodrat and deermice in addition to humans. The U.S. strains of *L. mexicana* have a unique genotype. Texan *L. mexicana* strains have been found to have unique polymorphisms different from the Mexican and South American strains at two positions of ITS2, i.e., A → C647 and T → C649. This unique genotype can be a very useful tool for identifying autochthonous *L. mexicana* in both animal reservoirs and humans in the USA [40,41,42].

## 5. Transmission Routes

*Leishmania* spp. are transmitted by three different routes among humans. First, they are naturally transmitted by sand fly vectors [43,44]. Second, they are vertically passed from mothers to offspring through the placenta during gestation. Third, they are horizontally spread out from infected donors to recipients by blood transfusion, an artificial transmission [3]. Here, we focus on transmission among U.S. dogs through routes other than sand fly vectors, which play a minimal, if any, role in leishmanial transmission in dog populations in the USA [9].

### 5.1. Vertical Transmission—Congenital Transmission

We present here lines of direct evidence for the congenital transmission of leishmaniosis from mothers to their offspring observed in the domestic dog. In a laboratory study, female beagles were infected with *L. infantum* parasites that were isolated from naturally infected Foxhounds in Virginia. These infected females were bred with a male chronically infected with the same parasite. Four puppies were delivered by Cesarean section. *Leishmania* DNAs were detected by PCR in internal organs/tissues of three puppies that were euthanized at birth; the remaining one was not tested due to being malformed and autolytic at delivery. This study unequivocally showed that *L. infantum* was congenitally transmitted from mother to fetus during pregnancy [45]. Further, two Foxhounds 19 months old in age tested positive for antibodies to *Leishmania* sp. by IFA. Their mother initially tested negative prior to breeding and became positive during pregnancy. The bitch died of leishmaniosis eight weeks after giving birth and two weeks after weaning the puppies. The two Foxhounds were diagnosed with *L. infantum* infections by microscopy, culture, immunohistochemistry and PCR [18]. This is the first confirmed report of vertical transmission of *L. infantum* from a naturally infected bitch to its puppies by placental transmission. Nevertheless, a slim chance of transmammary transmission cannot be completely ruled out since the two puppies had been breast fed for six weeks before their dam’s ultimate death of leishmaniosis. *Leishmania infantum* amastigotes have been detected in the mammary glands of infected dogs [46,47].

A Foxhound dam was brought into CO from KS to breed with a previously healthy Treeing Walker Coonhound sire reared in CO. The dam lived in CO for seven months in 2007 and then returned to KS. Eight months after returning, the dam was euthanized due to high titer antibodies to *Leishmania* spp. In the summer of 2007, the dam experienced epistaxis during the pregnancy and was found carrying 11 fetuses by ultrasound during a veterinary checkup. The dam gave birth to 10 puppies including four stillborn ones; one puppy died within a week and another one in a few weeks. None of these puppies were tested for *Leishmania* spp. Among the four survivors, one female returned to KS with the dam and was euthanized due to high antibody titer to *Leishmania* spp. The three males remained in CO. Among them one was diagnosed with *L. infantum* infection by microscopy, serology, and PCR at one year old. An additional male was euthanized at around three years old due to *Leishmania* infection, although it was not a well-documented diagnosis [19]. It is very likely these puppies were infected in utero during pregnancy. It has been shown that infected dogs may have a delay in showing clinical signs of leishmaniosis [48,49]. A naturally infected seven-year-old Foxhound was donated to a research laboratory. The dam that tested positive by IFA and qPCR for leishmanial kinetoplast DNA gave birth to 12 puppies. Eight were euthanized within 24 h after birth and the remaining four 12 weeks later. These puppies all tested positive for *Leishmania* spp. DNAs in at least one tissue/organ by the same qPCR except one puppy at each time point. The dam was euthanized 12 weeks after giving birth and tested positive for *Leishmania* spp. DNAs by PCR in multiple tissues/organs along with the placenta. This study shows direct evidence of congenital transmission and dissemination to internal organs in naturally infected Foxhounds [50]. Nevertheless, not all studies show such clear results. Eighteen canine dams of various breeds were diagnosed with *L. infantum* infection in Minas Gerais, Brazil. The breeds and numbers were Boxer—1, Cocker Spaniel—1, Doberman—3, Rottweiler—1, Siberian Husky—1 and mixed breed—11. They were all kept in a leishmaniosis research laboratory starting at gestation and beyond, along with their puppies (63 puppies in total including stillborn ones). Microscopy, culture and PCR were used to detect *Leishmania* parasites in various tissues/organs of the dams and offspring. All dams were confirmed *Leishmania* infected. Two of four placentas tested positive by PCR, and six milk samples from dams tested negative by PCR. However, none of the 63 offspring tested positive for *Leishmania* spp. in the spleen, liver, lymph nodes or bone marrow by the combination of the methods used. The authors concluded that vertical congenital infection is rare and plays a marginal role, if any at all, in spreading leishmaniosis in Brazil [51].

Differences exist between the last study and the others cited earlier. In addition to a dog’s breed, strains of *L. infantum* are different, i.e., Brazilian strains versus U.S. strains. The former is naturally transmitted by sand fly vectors, whereas the latter is not. It is plausible that U.S. strains of *L. infantum* have capably adapted to congenital transmission as a result of decades of spreading among Foxhounds without vectoring by sand flies. This can be addressed by comparative genomics of these strains to identify gene(s) contributing to vertical transmission.

Lastly, lines of evidence exist for molecular delineation of vertical transmission through clonal reproduction in U.S. Foxhound populations. In this case whole-genome sequencing and phylogenetic analyses were applied to seven *L. infantum* strains originating from U.S. Foxhounds. These parasites were determined to be imported to USA in late 19th and early 20th century by molecular clocking, much later than those brought to the New World approximately 500 years ago. Only clonal evolution existed without evidence of sexual reproduction in sand fly vectors. This clonal reproduction was further supported by the preservation of heterozygous sites across all seven U.S. strains [52].

### 5.2. Horizontal Transmission—Dog-to-Dog Transmission

#### 5.2.1. Blood Transfusion

Another likely route of *Leishmania* spp. transmission is horizontal transmission through blood transfusion. Several studies performed by various groups provide progressive evidence. First, canine blood donors from Natel, Brazil (a VL endemic area), were tested for anti-*L. donovani* antibodies with fucose–mannose ligand ELISA (FML-ELISA), which had been previously determined to have 100% sensitivity and 96% specificity. Among 1194 volunteer blood donors, 9% were antibody-positive. Further, at a follow-up performed five months later, four out of 27 donors with no clinical signs now developed clinical manifestations such as hepatosplenomegaly. One was confirmed as having *Leishmania* spp. infection by detecting amastigotes in the bone marrow [53]. The positive status of anti-*Leishmania* spp. antibody does not necessarily prove an individual is actively infected with these parasites. Among 21 clinically normal but antibody-positive individual blood donors by FML-ELISA, the presence of *Leishmania* spp. DNA by PCR and dot blot was confirmed in four and nine, respectively [54].

A serological survey was carried out in domestic dogs for *Leishmania* spp. transmission by blood transfusion. English Foxhound blood donors and recipients of various breeds were surveyed for antibodies to *Leishmania* spp. using IFA. None of the 25 recipients of packed red blood cells (pRBCs) from seronegative blood donors tested positive. In contrast, three of seven pRBC recipients of seropositive blood donors were positive. One of them was confirmed with *Leishmania* spp. infection by microscopic demonstration of amastigotes in aspirates of multi-tissues/organs and positive *Leishmania* culture. The parasite species was determined to be *L. infantum* [55]. This is the first confirmed case of *Leishmania* spp. transmission by blood transfusion. Additionally, transfusion of pRBCs has also been shown to be capable of transmitting *Leishmania* parasites to anemic recipient dogs. In this case, three out of seven recipient dogs given pRBCs from *Leishmania* spp.-positive donors were infected by the parasite. In contrast, all 33 dogs receiving pRBCs of negative donors were not [56]. Furthermore, hamsters that were inoculated peritoneally with whole blood or purified monocytes prepared from clinically normal donors but serologically positive for *Leishmania* antibodies established infections [57]. These lines of evidence confirm canine blood donors with a *Leishmania*-positive status, even without clinical manifestations, can transmit the parasites to blood transfusion recipients, which greatly solidifies a similar notion in humans [3].

#### 5.2.2. Venereal Transmission from Male to Female Dogs

In a controlled laboratory experiment, 12 *Leishmania*-free bitches were mated with naturally *L*. *infantum*-infected males. By the end of the study, 165 days after the last copulation, three females tested positive serologically and six tested positive by PCR. In contrast, the two control bitches maintained under the same laboratory conditions but not mated remained negative throughout the experiment. These findings clearly demonstrate sexual transmission from *Leishmania*-positive male to *Leishmania*-negative female dogs [58]. However, its epidemiological relevance in the USA, particularly within Foxhound populations remains uncertain.

#### 5.2.3. Direct Dog-to-Dog Transmission Through Bites/Wounds

Two female Jack Russel Terriers, one born in October 2007 (dog A) and the other born in 2009 (dog B), were kept in the same household in Stolberg (Rhineland), a non-leishmaniasis endemic area, between 2011 and 2012. Dog B received bite wounds from dog A. Dog B experienced vomiting, diarrhea, edema of the legs and head, apathy, and anorexia soon after being bitten by dog A in January 2012. It was diagnosed with leishmaniosis by positive serology and microscopic demonstration of *Leishmania* amastigotes although PCR failed to detect *Leishmania* DNA in blood. It was euthanized due to poor prognosis. Dog A which had never traveled to an endemic area became lethargic and inactive in December 2015 and was diagnosed by serology demonstrating antibodies to *L. infantum*. The authors concluded this was the first case of dog-to-dog transmission of naturally infected leishmaniosis through biting wounds [59]. This transmission route may also play an important role in *Leishmania* transmission among U.S. Foxhound populations alongside vertical congenital transmission.

## 6. Concluding Remarks

Texas is the only U.S. state that has designated leishmaniasis reportable since 2007. The disease is certainly underdiagnosed and underreported in the state [60,61]. Regarding the unique situation of autochthonous infections by *L*. *infantum* in the U.S. Foxhounds, the confirmed transmission routes are vertical transmission from bitches to puppies through the transplacental route and horizontal transmission through blood transfusion, venereal transmission from male to female and bite wound contamination. Further, *L*. *infantum* is the dominant species of imported canine leishmaniosis [62]. It is worth pointing out that elimination of *L*. *infantum*-positive dogs such as culling in Brazil is unsuccessful in the control of human leishmaniasis [63]. These canine hosts certainly pose risk to humans, although how important a role they play in *Leishmania* endemicity in the USA remains to be seen. On the other hand, wildlife, mainly woodrats and deermice, have been confirmed as natural reservoirs in Texas and Arizona. More research on these reservoirs as well as on other wildlife such as wild canids, especially in southern states, is urgently needed. This information is critical for developing strategies to control and manage *Leishmania* spp. and leishmaniasis by expanding reservoir and vector surveillance through a One-Health approach in the USA.

## Figures and Tables

**Table 2:** Autochthonous feline cases of leishmaniosis in USA (open cells indicate no data available).

Year	State	Age	Sex	Breed	Clinical Signs	Diagnostic Methods *	*Leishmania* sp.	Treatment (/F/H/LTF) ^¥^	References
1984	TX	6	M	Long-haired domestic cat	Four large and several tumors on left pinna	AI, C, IP, M	*L. mexicana*	Pinnectomy (F), euthanasia	[21,22]
2004 2004 2006 2006 2007 2007 2008 2008	TX TX TX TX TX TX TX TX	3 11 3 4.5 5 3 1	M M F M F M M F	Mixed Mixed Mixed Mixed Mixed Mixed Mixed Mixed	Skin nodule Skin nodule Skin nodule Skin nodule Skin nodule Skin nodule Skin nodule Skin nodule	M D, PCR, M M D, PCR, M M D, PCR, M D, PCR, M D, PCR, M	*L. mexicana* *L. mexicana* *L. mexicana* *L. mexicana* *L. mexicana*	LTF LTF Surgery (H) Surgery (F), euthanasia Allopurinol (LTF) Surgery (H) Surgery (H) LTF	[24]
2021 ^+^	TX	6	M	Short-haired domestic cat	Non-healing wounds on the right pinna and the right tarsus	D, PCR, M	*L. mexicana*	Marbofloxacin (F), artemisinin (F)	[23]

*: AI: animal infection; C: culture; D: DNA sequencing; IP: isoenzyme profile; M: microscopy of amastigotes; PCR: polymerase chain reaction. ^¥^: F: failure; H: healed; LTF: lost to follow-up. ^+^: Publication year.

## Data Availability

No new data were created or analyzed in this study. Data sharing is not applicable to this article.
